# Effect of Superheated Steam Treatment on the Lipid Stability of Dried Whole Wheat Noodles during Storage

**DOI:** 10.3390/foods10061348

**Published:** 2021-06-11

**Authors:** Wan-Ting Jia, Zhen Yang, Xiao-Na Guo, Ke-Xue Zhu

**Affiliations:** State Key Laboratory of Food Science and Technology, School of Food Science and Technology, Jiangnan University, 1800 Lihu Avenue, Wuxi 214122, China; 6180112021@stu.jiangnan.edu.cn (W.-T.J.); zhen.yang@jiangnan.edu.cn (Z.Y.); xiaonaguo@jiangnan.edu.cn (X.-N.G.)

**Keywords:** dried whole wheat noodles, superheated steam treatment, lipid stability, enzyme activity, tocopherols, volatile compounds

## Abstract

Dried whole wheat noodles (DWWN) are a kind of nutritious convenience food with broad market prospects. However, due to the presence of high content of unsaturated fatty acids (UFAs) and lipid degrading enzymes, the shelf life and edible quality of DWWN are easily affected. This study explored the effect of superheated steam treatment (SST-155 °C-10 s, SST-170 °C-10 s, SST-190 °C-5 s) on the lipid stability of DWWN. The lipase, lipoxygenase and peroxidase of the DWWN treated with superheated steam were completely passivated during storage. After 12 weeks of storage, the fatty acid value of DWWN increased by 35.1, 17.9, 15.9, 24.6 mg NaOH/100 g in the groups of control, SST-155 °C-10 s, SST-170 °C-10 s, SST-190 °C-5 s, respectively; whereas the content of UFAs decreased by 13.5%, 6.8%, 5.4%, and 2.7%, respectively. The content of 2-pentylfuran in the SST-155 °C-10 s, SST-170 °C-10 s, SST-190 °C-5 s group was 0.7, 0.6, and 0.4-fold than that of the control group, respectively. In addition, the total tocopherol and total volatile compounds of the SST-190 °C-5 s group were 2.4 and 0.7-fold than that of the control group, respectively. Therefore, SST should be a new technology that can improve the lipid stability of DWWN.

## 1. Introduction

Intake of dietary fiber and phytochemicals can help reduce the risk of chronic diseases (such as cancer, diabetes, and cardiovascular disease) [1,2]. In recent years, whole-grain foods such as whole wheat and coarse cereals have attracted more attention because they are rich in dietary fiber, minerals and phytochemicals [3,4,5]. Whole wheat is rich in tocols (tocopherols and tocotrienols), whose content is about 27.6–79.7 μg/g flour [6]. Tocols are the most important compounds with antioxidant activity in vegetable oils. It can not only inhibit the peroxidation of unsaturated fatty acids (UFAs) and other compounds [7], but also prevent neurological diseases, induce immune responses and lower cholesterol [8]. Noodles are one of the most popular staple foods in Asian countries, and their raw materials account for about 20–50% of the total wheat flour [4]. Dried noodles originated in China, with the characteristics of easy storage and convenient eating, and a high degree of industrialization. In addition, dried whole wheat noodles (DWWN) meet the needs of consumers who desire healthy and low-calorie foods [4,9].

However, one of the main factors affecting the development of DWWN industry is the short shelf-life. The content of UFAs and the activity of lipid degrading enzymes in DWWN are higher than that of ordinary dried noodles due to the presence of bran and germ [2,10]. Lipid degrading enzymes can act on UFAs to produce a series of lipid hydrolysis and oxidation reactions, resulting in bad odors, which affects the nutritional quality and edible quality of DWWN [10,11]. Moreover, the rate of lipid hydrolysis and oxidation was significantly correlated with the activity of lipid degrading enzymes such as lipase, lipoxygenase (LOX) and peroxidase (POD) [2,10,11]. Lipase generally acts on the ester bonds of triglycerides and releases free fatty acids (FFAs) [11]. LOX catalyzes the enzymatic oxidation of UFAs to form hydroperoxides [12]. POD can decompose hydroperoxides into small molecular compounds, such as aldehydes, ketones, and organic acids [13]. 

At present, inactivating the lipase and LOX in whole wheat flour (WWF) is an effective way to extend the shelf life of DWWN. Some processing methods including hot air treatment, steam treatment, microwave and irradiation have been widely used to stabilize WWF and wheat bran [2,13,14]. However, these traditional treatment methods have some limitations, such as incomplete enzyme inactivation, promotion of auto-oxidation, impaired nutritional quality [15,16]. In recent years, superheated steam treatment (SST) has appeared as an emerging technology to inactivate enzymes and decontaminate microorganisms [17]. SST is the dry steam generated by adding sensible thermal energy to wet saturated steam, which makes the steam temperature higher than the corresponding boiling point or saturation point in a certain pressure [18]. If only its temperature is kept above the saturation point, it has the ability to absorb moisture and is suitable for processing dry food materials [17]. Superheated steam has a large heat transfer coefficient, high thermal efficiency, and high temperature, resulting in a short processing time and energy saving (steam can be recycled) [2,19,20]. Studies have shown that SST is an effective and feasible method of stabilization, which can effectively reduce the activities of lipase and LOX, while having little effect on phenols, flavonoids and antioxidant activity [2,15,16,17]. This is because superheated steam treatment can provide an oxygen free environment, which is conducive to inhibiting lipid oxidation [15]. Superheated steam has been used to inactivate enzymes and dry cereals such as wheat, brown rice and oats [21]. However, the effect of SST on the lipid stability of DWWN has not been reported yet.

The aim of the present study was to explore the effect of SST-processed WWF on the lipid stability of DWWN during storage. This study used SST-processed WWF to prepare DWWN, and determined its enzyme activity, fatty acid value, fatty acid composition, tocols content and volatile compounds during storage. The results of this study indicated that SST could significantly improve the lipid stability of DWWN during storage. Therefore, this study proposed a new method to improve the lipid stability of DWWN, which provided a theoretical basis for industrial production.

## 2. Materials and Methods

### 2.1. Reagents and Raw Material

Guaiacol and linoleic acid were purchased from Macklin Biochemical Co., Ltd. (Shanghai, China), and 4-nitrophenyl caprylate was purchased from Alfa Aesar Chemical Co., Ltd. (Shanghai, China). The α-, β-, γ-, and δ-tocopherol standards, and methyl nonadecylate were purchased from Sigma-Aldrich Co., Ltd. (St. Louis, MO, USA). Tocomin SupraBio (containing four tocopherols and four tocotrienols) was purchased from Healthy Origins Inc. (Perak, Malaysia). All other reagents, including isopropanol, heptane and 1,4-dioxane were purchased from Merck KGaA (Darmstadt, Germany). The reagents were of analytical grade, and the solvents of high-performance liquid chromatography (HPLC) grade were purchased from Sinopharm Chemical Reagent Co., Ltd. (Shanghai, China).

Raw materials including wheat bran and straight flour were provided by Yihai Kerry Grain, Oil and Food Industry Co., Ltd. (Dongguan, Guangdong Province, China). The wheat bran was ground by a low-temperature grinder (Mode SES-01, Wuxi Hepu Light Industrial Equipment Technology Co., Ltd., Jiangsu, China) and passed through an 80 mesh (180 μm) standard experimental sieve. The wheat bran and straight flour were reconstituted into WWF according to the yield of real-time display and automatic calculation of the factory. The compositions of WWF were characterized according to the American Association of Cereal Chemists (AACC 2000) methods [22], including 12.85% moisture (AACC method 44-01.01), 1.64% fat (AACC method 30-25.01), 1.67% ash (AACC method 08-12.01), 10.84% protein (AACC method 39-11.01). The raw materials stored in the refrigerator at −18 °C before analysis.

### 2.2. Superheated Steam Treatment of Whole Wheat Flour

The WWF was treated using superheated steam powder sterilization equipment (Mode WS-FMD15, Jiangsu Wanchuang Sterilization Equipment Technology Co., Ltd., Jiangsu, China). The conditions and process of SST were as follows: when superheated steam reached set temperature, the 500 g of WWF was put into the feeding system. Then the steam stirring paddle drove the WWF to rotate at a speed of 1000 r/s. Meanwhile, the superheated steam with the volume flow of 22 m^3^/h was fed into the processing chamber, so that the WWF suspended in the high-speed flowing superheated steam. The contact between WWF and superheated steam was uniform during SST. After SST, the WWF was separated from the steam through the separator equipped in the processing chamber. The separated WWF was cooled rapidly by a condenser. The parameters of SST were set at 155 °C-10 s, 170 °C-10 s, 190 °C-5 s. After the SST processing, the treated WWF was stored in the refrigerator at −18 °C until use.

### 2.3. Preparation and Storage Experiment of Dried Whole Wheat Noodles

The dried noodles were prepared according to the method of Yu et al. [9] with some modifications. The DWWN formula consisted of 400 g of WWF, 108 mL of distilled water and 4 g of salt. Ingredients were mixed using a vacuum mixer (HWJZ-5, Nanjing, Jiangsu Province, China) for 7 min (vacuum degree was −0.08 MPa). The mixed dough was put into a sealed bag and left to rest for 30 min at 25 °C and 75% relative humidity (RH). Then, the dough was passed through a small noodles machine (JMTD-168/140, Beijing, China) to get 1mm thick and wide wet noodles. Finally, DWWN was obtained by using a drying equipment for dried noodles (SYT-030, Beijing, China) to dry in stages for 230 min. The drying process of dried noodles included five stages, and the temperature, RH and time of each stage were different: Stage I: 35 °C, 80% RH, 35 min; Stage II: 40 °C, 70% RH, 35 min; Stage III: 45 °C, 60% RH, 85 min; Stage IV: 40 °C, 60% RH, 35 min; Stage V: 30 °C, 60% RH, 40 min.

The DWWN (the length was about 20 cm) was packed in vacuum bags and sealed using a heat-sealing machine (D2 300/5SA, Dongguan, Guangdong Province, China), and then stored at 40 °C and 75% RH in an incubator for storage experiment. The thickness of the vacuum packaging bag was 0.2 mm, which was made of polyethylene terephthalate and polyethylene composite processing. The samples were taken and analyzed in an interval of 3 weeks. In addition, a portion of DWWN was cut into 1 cm length and stored in dark-brown headspace bottles at the same condition to determine volatile compounds in order to avoid the loss of odor. The DWWN made from WWF without SST was used as a control group. The DWWN were crushed by high-speed universal crusher (FW100, Shanghai, China), and then passed through an 80 mesh (180 μm) standard experimental sieve to obtain dried noodles flour.

### 2.4. Determination of Lipase, Lipoxygenase and Peroxidase Activity

#### 2.4.1. Determination of Lipase Activity

The lipase activity of DWWN was determined referring to the method of Cai et al. [23] with some modifications. The 2 g of dried noodles flour was mixed with 10 mL of Tris-HCl (pH 8.0, 50 mM) buffer using a vortex, then the mixture was placed in an ice bath at 4 °C for 30 min and shaken intermittently during the period. After that, the mixture was centrifuged with a centrifuge (H2050R, Xiangyi Laboratory Instrument Development Co., Ltd., Hunan, China) for 10 min (4 °C, 10,275× *g*), the supernatant was filtered through a 0.45 μm filter membrane to obtain the crude enzyme solution. To start the reaction, 1.78 mL of Tris-HCl buffer, 20 μL of 10 mM 4-nitrophenol caprylate substrate and 200 μL of crude enzyme solution were added into the enzyme label plate in sequence. The absorbance change of the reaction solution was determined within 3 min at 37 °C and 405 nm using a microplate reader (Bio Tek Epoch 2, Berten Instrument Co., Ltd., Burlington, VT, USA). Enzyme activity was expressed as the absorbance of the reaction system increases by 0.01 to 1 U per minute, and finally the unit was converted to U/g·min^−1^.

#### 2.4.2. Determination of Lipoxygenase Activity

The lipoxygenase activity of DWWN was determined referring to the research method of Cato et al. [24] with some modification. The 1 g of dried noodles flour was mixed with 10 mL of phosphate buffer solution (pH 7.5, 0.1 mol/L) using a vortex, then the mixture was extracted with ice bath for 30 min. After that, the mixture was centrifuged with a centrifuge for 20 min (4 °C, 10,275× *g*), then the supernatant solution was filtered through a 0.45 μm filter membrane to obtain the crude enzyme solution. The substrate solution was prepared by dissolving 99.93 mg of linoleic acid standard into 10 mL of absolute ethanol in a nitrogen atmosphere. Then, 80 µL of tween 20 was added to 7.1 mL of the prepared substrate solution. A rotary evaporator was used to remove the ethanol, and the residue was dissolved in 100 mL of 0.05 mol/L Na_2_HPO_4_. Then the pH was adjusted using 1 mol/L NaOH to a final value of pH = 9. The concentrations of linoleic acid and tween-20 in the substrate solution were 2.53 mmol/L and 0.08% (*w*/*v*), respectively. To start the reaction, 2.89 mL acetate buffer (0.05 mol/L, pH = 5.5), 90 μL of substrate solution and 20 μL enzyme solution was added successively, and the absorbance change of the reaction system was determined within 3 min at 234 nm using a spectrophotometer (TU-1810, Puxi General Instrument Co., Ltd., Beijing, China). Enzyme activity was expressed as the absorbance of the reaction system increases by 0.01 to 1 U per minute, and finally the unit was converted to U/g·min^−1^.

#### 2.4.3. Determination of Peroxidase Activity

The peroxidase activity of DWWN was determined referring to the research method of Sessa et al. [25] and Jiang et al. [26] with slight modifications. The 2 g of dried noodles flour was mixed with 10 mL of distilled water, then the mixture was placed at room temperature for 30 min and shaken intermittently during the period. Then, the mixture was centrifuged for 10 min (25 °C, 10,275× *g*), and the supernatant solution was the enzyme extract. The mixture of H_2_O_2_ solution (30% H_2_O_2_: water = 1:29, *v*/*v*) and guaiacol solution (1% guaiacol:96% ethanol = 1:99, *v*/*v*) were premixed at the ratio of 1:1. After that, the 600 μL of the mixture and 1.2 mL of the enzyme extract were added to the enzyme label plate to start the reaction. The absorbance change of the reaction system was determined at 470 nm within 3 min using a microplate reader. Enzyme activity was expressed as the absorbance of the reaction system increases by 0.01 to 1U per minute, and finally the unit was converted to U/g·min^−1^.

### 2.5. Extraction of Lipids from Dried Whole Wheat Noodles

The extraction of lipids in DWWN was carried out according to the method of Yao et al. [27] using an accelerated solvent extraction (ASE) instrument (DIONEX ASE350, Thermo Fisher Scientific Co., Ltd., Waltham, MA, USA). The 1 g of dried noodles flour was mixed with the same amount of quartz sand in an 11 mL of extraction cell, then filled the cells with quartz sand. Lipid extraction was carried out with ASE under the condition of 1000 psi pressure and 100 °C temperature with a preheating time of 5 min, and 2 static extraction cycles of 10 min. The extraction solvent was acetone. Then, the extract was transferred to a round-bottomed flask, and evaporated under reduced pressure using a rotary evaporator at 40 °C. Finally, the dried lipids were dissolved in 10 mL of heptane for further analysis.

### 2.6. Determination of Fatty Acid Composition

The fatty acid composition of DWWN was determined referring to the method of Shin et al. [28]. The content of lipids and fatty acid composition in DWWN were determined by a gas chromatograph (GC-2010PLUS, Shimadzu Co., Ltd., Tokyo, Japan) coupled with a flame ionization detector (GC-FID). An external standard method was used to quantify the fatty acid methyl ester (the external standard was methyl nonadecylate). The content of lipids was calculated by the sum of fatty acid methyl ester. The 2 mL of the lipid solution prepared in Section 2.5 was transferred into 10 mL clean glass tubes and dried with nitrogen. Then 2 mL of 2% NaOH-CH_3_OH solution was added to dissolve the oil droplets, and the solution was heated at 65 °C for 30 min until the oil droplets disappeared. After cooling to room temperature, 2 mL of 14% BF_3_-CH_3_OH solution was added and the above heating process was repeated. After cooling, 2 mL of heptane was added and the lipids were extracted by shaking for 5 min. Then, 2 mL saturated NaCl solution was added and allowed to stand for 2 h. After that, the upper organic phase was transferred into a test tube containing 0.5 g of anhydrous sodium sulfate and left for 30 min. Finally, the solution was collected to filter through a 0.22 μm filter membrane for GC analysis.

The GC conditions were set as follows: the chromatographic column was DB-WAX capillary column (30 m × 0.25 mm × 0.25 μm); the temperature of detector and injector was 250 °C, the carrier gas was high-purity nitrogen with a flow rate of 3 mL/min; the temperature rising program was as follows: the temperature was from 150 °C to 190 °C at the rate of 5 °C/min and maintained for 2 min, and then to 240 °C at the rate of 5 °C/min and maintained for 10 min.

### 2.7. Determination of Tocopherols and Tocotrienols Content

The tocopherols and tocotrienols content of DWWN was determined referring to the method of Lampi et al. [6] with some modifications. The α-, β-, γ- and δ-tocopherol standards were dissolved and preserved in heptane, the content of tocopherols in the samples were quantified by making calibration curves with the α-, β-, γ- and δ- tocopherol standards in the range of 2–150 ng/injection. The tocotrienols in samples were identified by the commercially purchased Tocomin SupraBio product containing tocopherols and tocotrienols. Each of the tocotrienol was quantified with its corresponding calibration curve of tocopherol standard. The concentration and purity of the standard storage solution were checked with spectrophotometer in an interval of 4 weeks. 

The content of tocopherols and tocotrienols in DWWN was determined by normal phase high-performance liquid chromatograph (Waters1525, Waters Co., Ltd., Milford, MA, USA) coupled with a fluorescence detector (NP-HPLC-FLD). The lipid solution prepared in Section 2.5 was pipetted through a 0.22 μm filter membrane into a liquid phase bottle for analysis. The GC conditions were set as follows: the chromatographic column was Inertsil silica column (30 cm × 3.9 mm, 5 μm; Varian Inc.; Palo Alto, CA, USA), the mobile phase was heptane:1,4-dioxane = 97:3, the flow rate of the mobile phase was 1 mL/min with isocratic elution at a column temperature of 30 °C. The excitation wavelength (λ_ex_) of the FLD was 292 nm, and the emission wavelength (λ_em_) was 325 nm.

### 2.8. Characterization of Volatile Compounds

The volatile compounds of DWWN were determined referring to the method of Paradiso et al. [29] with some modifications. Volatile compounds were determined by headspace solid-phase microextraction gas chromatograph coupled with mass spectrometer (HS-SPME-GC-MS) (TSQ Quantum XLS, Thermo Fisher Scientific Co., Ltd., Waltham, MA, USA). The extraction head was inserted into the 20 mL of headspace bottle containing 2 g of DWWN, and extracted for 30 min at 60 °C. After extraction, the sample was injected quickly and the resolution time was 5 min. The GC-MS spectrum was matched and searched with Wiley 7N (Wiley Registry™ of Mass Spectral Data, 7th Edition, Scientific Instrument Services Inc., Ringoes, NJ, USA) database. 

The GC conditions were set as follows: the flow rate of helium was 0.8 mL/min and splitless injection, and the chromatographic column was capillary column DB-WAX (30 m × 0.25 mm × 0.25 μm). The heating program was as follows: the initial temperature was kept at 40 °C for 4 min, the temperature was increased to 70 °C at the rate of 5 °C/min, then the temperature was increased to 250 °C at 10 °C/min and maintained for 8 min. The MS conditions are as follows: the ionization energy was 70 eV, ion source temperature was 200 °C and ions were scanned from 33 to 450 amu. The analysis was calibrated using an alkane solution (n-butane to n-hexadecane). Total ion counts were used for quantifying the amounts of compounds, and the content of volatile compounds in the sample was expressed by peak area and relative content.

### 2.9. Determination of Fatty Acid Value

Fatty acid value of DWWN was determined by a titration of Zhao et al. [30] with slight modifications. Firstly, 5.0 g of dried noodles flour was mixed with 30 mL of 95% ethanol and extracted by shaking for 1 h. After centrifugation for 10 min (25 °C, 10275 g), the supernatant was collected and 20 mL of which was titrated with 0.05 mol/L NaOH-ethanol solution. The end point of titration was pink appeared and lasted for 30 s with phenolphthalein (1%, *w*/*v*) as an indicator. The fatty acid value (mg NaOH/100 g) of DWWN was calculated according to the Equation (1).

Fatty acid value = 600000 × (*V*_1_ − *V*_0_) × *c*/*m* × (100 − *w*) (1)

where *V*_1_ = volume of sample titration (mL), *V*_0_ = volume of blank titration (mL), *c* = normality of titrant (mol/L), *m* = weight of sample (g), and *w* = moisture of sample (%).

### 2.10. Statistical Analysis

Analysis of one-way analysis of variance (ANOVA) with Tukey’s test at a 95% confidence level using SPSS version 22 (IBM SPSS statistics, International Business Machines Co., Ltd., Armonk, NY, USA). The influencing factors were SST-processed parameters and storage time. All results were expressed as mean ± standard deviation (SD) of the least three replicates. The charts were drawn using origin 2018 (version pro9.1, Originlab Co., Ltd., Northampton, MA, USA).

## 3. Results and Discussion

### 3.1. Changes of Enzyme Activity in Dried Whole Wheat Noodles during Storage

According to the study of Guo et al. [2], when the temperature of SST was 155/170 °C and the treatment time was more than 10 s, or when the treatment temperature was 190 °C and the treatment time was more than 5 s, the activities of lipase and LOX in WWF could not be detected. Therefore, three parameters of SST-155 °C-10 s, SST-170 °C-10 s, and SST-190 °C-5 s were used to process WWF in this study. 

As shown in Table 1, Table 2 and Table 3, the activities of lipase and POD were not detected in DWWN made from SST-processed WWF. In addition, compared with the control DWWN, the LOX activity was reduced by 67.6%, 69.3%, 71.3% in the SST-155 °C-10 s, SST-170 °C-10 s, and SST-190 °C-5 s group, respectively. In previous studies, the effectiveness of SST to inactivate enzymes was also reported. Hu et al. [15] found that the POD of wheat bran was completely inactivated after SST at 170 °C for 7 min. Wu et al. [31] found that the POD was completely inactivated after the lightly milled rice was processed by SST at 110/120 °C for 3.5/2.5 min. The activity of POD was used as an indicator of the heat treatment effect [31,32]. On the one hand, the inactivation of enzymes by SST may be due to the high temperature treatment destroyed the structure of the enzymes, leading to enzyme denaturation, which made lipase and LOX “inactivated” or “passivated” [33]. On the other hand, it may be because the high temperature treatment destroyed the water environment of enzyme-catalyzed reactions [34].

**Figure 1:**
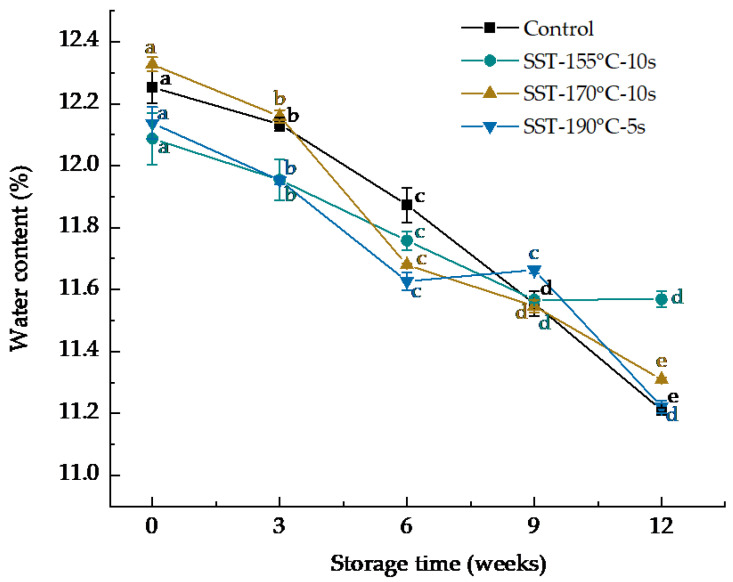
Effect of superheated steam treatment on moisture content of dried whole wheat noodles during storage. The lowercase letters indicate the significant difference of the same sample in different storage time at *p* < 0.05. SST: superheated steam treatment.

During storage, the activities of lipase, LOX, and POD were not detected in all the SST groups. The initial activities of these enzymes were high in the control group, whereas they decreased significantly during storage (*p* < 0.05), i.e., by 54.5% (Table 1) of lipase activity, by 93% of LOX activity (Table 2) and by 58.7% of POD activity (Table 3) after 12 weeks of storage. This may be due to the fact that lipase hydrolysis of lipids resulted in the accumulation of FFAs, which in turn inhibited lipase activity, and the storage temperature (40 °C) deviated from the optimal temperature of lipase [35,36]. It may also be due to the significant decrease (*p* < 0.05) in moisture content during storage (Figure 1), because moisture can change the fluidity of the substrate and the reactant or the active conformation of the enzyme [37]. The decrease in moisture content of all sample groups during storage may be caused by storage temperature (40 °C); on the other hand, it may be because the vacuum bags did not completely block the moisture exchange between dried noodles and the outside world [38]. Xu et al. [39] found that lipase could hydrolyze lipids to generate FFAs, and lipase activity was significantly related to water activity. In conclusion, the SST significantly inhibited the activities of lipase, LOX and POD of DWWN during storage.

### 3.2. Changes of Fatty Acid Value in Dried Whole Wheat Noodles Storage

**Figure 2:**
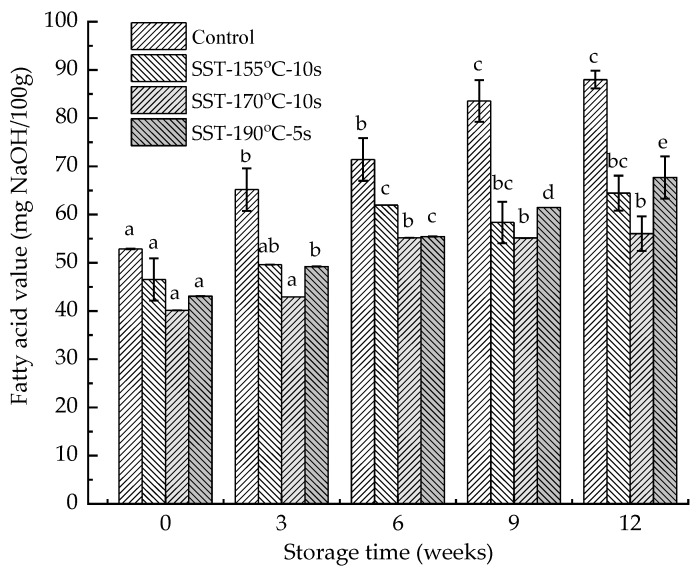
Effect of superheated steam treatment on fatty acid value of dried whole wheat noodles during storage. The lowercase letters in the column chart indicate the significant difference of the same sample in different storage time at *p* < 0.05. SST: superheated steam treatment.

The fatty acid value of DWWN in the control group was 52.9 mg NaOH/100 g (Figure 2). The fatty acid value of DWWN made from SST-processed WWF was slightly decreased. For example, the values in SST-155 °C-10 s, SST-170 °C-10 s, SST-190 °C-5 s group decreased by 11.9%, 24.1%, and 18.5%, respectively (Figure 2). The hydrolysis of triacylglycerol by lipase led to the accumulation of FFAs. The UFAs in FFAs can undergo enzymatic oxidation or auto-oxidation to form hydroperoxides. Therefore, FFAs were not only the products of enzymatic hydrolysis, but also the reaction substrates of enzymatic oxidation/auto-oxidation [40,41]. The fatty acid value of DWWN decreased after SST, which may be due to the high temperature during the heat treatment that promoted the oxidation of UFAs in FFAs, or the inactivation of lipase by SST that inhibited the hydrolysis of lipids in the process of dried noodles [42].

As shown in Figure 2, the fatty acid value of the control group increased significantly during storage (*p* < 0.05), while the values in SST groups increased slowly. After 12 weeks of storage, the control group, SST-155 °C-10 s, SST-170 °C-10 s, SST-190 °C-5 s group increased by 35.1, 17.9, 15.9, 24.6 mg NaOH/100 g, respectively. The accumulation rate of FFAs was closely correlated with lipase activity [42]. In the SST groups, the lipase of DWWN was passivated but the fatty acid value still increased. This may be because the residual lipase activity unable to be detected due to the limitation of the lipase determination method, or it may be because the lipid is under the action of thermodynamics hydrolyzed to produce FFAs (the storage temperature of DWWN was 40 °C) [43]. To conclude, the result showed that the SST can effectively inhibit the growth of fatty acid value during the production process and storage period of DWWN.

### 3.3. Changes of Fatty Acid Composition in Dried Whole Wheat Noodles during Storage

Changes of the fatty acid composition in the control group and the SST groups were shown in Table 4. The lipid content of control DWWN was 12.7 mg/g. The fatty acids detected by GC-FID mainly included saturated fatty acids (SFAs, including palmitic acid, stearic acid) and UFAs (oleic acid, linoleic acid and linolenic acid), of which UFAs accounted for up to 77.3%. Linoleic acid (46.4%) had the highest content among UFAs in DWWN, followed by oleic acid (20.6%) and linolenic acid (10.3%). The results were consistent with Obadi et al. [44] and Mufari et al. [7]. As shown in Table 4, the content of UFAs in DWWN made from SST-processed WWF was significantly higher than that of control DWWN (*p* < 0.05), and there was no significant difference for SFAs (*p* > 0.05). At the initial stage of storage, the content of UFAs in the SST groups was 1.2-fold than that of the control group. This indicated that SST can significantly prevent the degradation of lipids during production of DWWN, whereas did not change its fatty acid composition.

The content of UFAs in all sample groups decreased during storage, among which the content of UFAs in the control group decreased significantly (*p* < 0.05), and there was no significant change in the SST-190 °C-5 s group (*p >* 0.05). After 12 weeks of storage, the content of UFAs in the control group, the SST-155 °C-10 s, SST-170 °C-10 s, SST-190 °C-5 s group decreased by 13.5%, 6.8%, 5.4%, 2.7%, respectively. Linoleic acid decreased the most in all sample groups. This was because linoleic acid accounted for the highest proportion of UFAs, and more prone to oxidation than mono-unsaturated fatty acids such as oleic acid [45]. Among the three SST groups, the SST-190 °C-5 s group had the least degradation of UFAs after storage for 12 weeks, but the increase in fatty acid value was the largest (Figure 2). This may be because although the enzymatic oxidation pathways of all SST groups were blocked due to LOX inactivation, the auto-oxidation pathway rate in the SST-190 °C-5 s group was lower than other SST groups due to the shorter treatment time (5 s), resulting in the accumulation of FFAs [6,26,46]. As a result, SST can effectively inhibit the degradation of UFAs during the production process and storage of DWWN, and the SST-190 °C-5 s group had the best inhibitory effect.

### 3.4. Changes of Tocopherols and Tocotrienols Content in Dried Whole Wheat Noodles during Storage

The content of tocols, including two tocopherols (α- and β-tocopherol) and four tocotrienols (α-, β-, γ- and δ-tocotrienol), in control group of DWWN was 7.7 μg/g (Table 5), in which α-tocopherol had the highest content, with a content of 3.1 μg/g, followed by β-tocotrienol, with a content of 1.9 μg/g. In general, the content of tocotrienols in DWWN was higher than tocopherols, which was consistent with the result of Lampi et al. [6] and Shadyro et al. [47]. As natural antioxidants, tocols can act as a hydrogen donor and combine with hydroperoxide radicals (ROO•) to form hydrogen peroxide, which can prevent lipid autoxidation by inhibiting the peroxidation of UFAs and other compounds [7,48]. The combination rate of tocols and ROO• is far higher than that of UFAs and ROO•. One tocopherol molecule can even protect 10^4^–10^9^ UFA molecules [49,50]. Therefore, the content of tocols can reflect the degree of lipid auto-oxidation of DWWN during storage. The tocols content of DWWN made from SST-processed WWF was significantly higher than that of control DWWN (*p* < 0.05), and the content in the SST-155 °C -10 s, SST-170 °C-10 s, SST-190 °C-5 s group was 1.4, 1.6 and 1.6-fold than that of the control group. This indicated that the SST can effectively inhibit the consumption of tocols during production of DWWN.

The content of tocopherols and tocotrienols in DWWN decreased significantly (*p* < 0.05) during storage. After 12 weeks of storage, the control group, SST-155 °C -10 s, SST-170 °C-10 s, SST-190 °C-5 s group decreased by 3.2, 6.1, 6.3, 1.3 μg/g, respectively. The high reduction of tocols in groups of SST-155 °C-10 s and SST-170 °C-10 s may be due to the long treatment time, and also the high temperature promoted lipid auto-oxidation and thus produced more free radicals. The content of tocols decreases by scavenging free radicals during storage [51]. Besides, the SST-155 °C-10 s and SST-170 °C-10 s group had less reduction in UFA during storage (Table 4), most likely because the tocols were consumed in the reaction, which slowed the lipid degradation process. After storage for 12 weeks, the tocols content of the SST-155 °C-10 s, SST-170 °C-10 s, SST-190 °C-5 s group was 1.0, 1.3 and 2.4-fold compared to the control group, of which the tocols content in SST-190 °C-5 s was the highest. It indicated that the tocopherols and tocotrienols of the SST-190 °C-5 s group were the most stable during storage.

### 3.5. Changes of Volatile Compounds in Dried Whole Wheat Noodles during Storage

**Figure 3:**
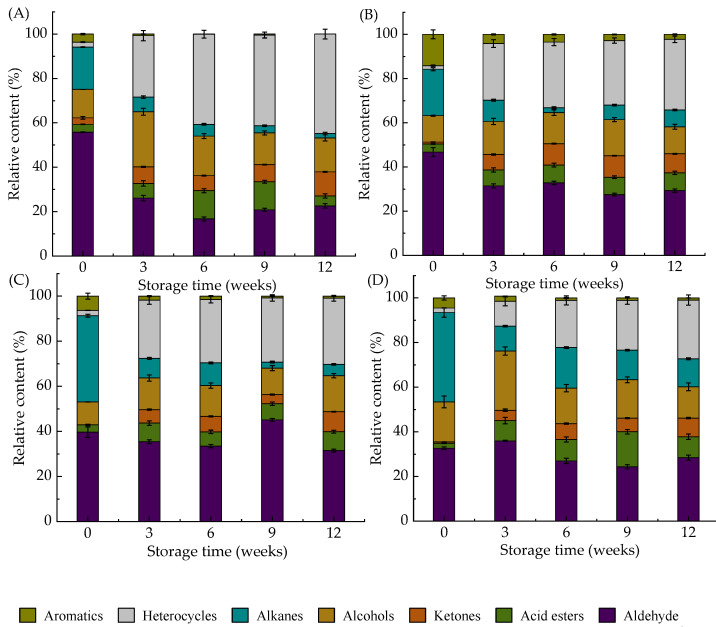
Effect of superheated steam treatment on relative content of volatile compounds in dried whole wheat noodles during storage. (**A**) Control, (**B**) SST at 155 °C for 10 s, (**C**): SST at 170 °C for 10 s, (**D**) SST at 190 °C for 5 s. SST: superheated steam treatment.

As shown in Figure 3, the volatile compounds detected in DWWN mainly consisted of aldehydes, ketones, acid esters, alcohols, alkanes, heterocyclics and aromatics. Volatile compounds are mostly the products of lipid oxidation. Lipid oxidation produces hydroperoxides, which are chemically unstable and easily broken down into smaller volatiles through different pathways [52]. The relative content and type of volatile compounds in DWWN were affected by the heat treatment and storage time. As the storage progresses, the relative content of alkanes and aromatics in all sample groups decreased, while the relative content of alcohols, acid esters, ketones, aldehydes, and heterocyclics increased (Figure 3). The relative content of aldehydes in the control group decreased, while the content of heterocyclics increased (Figure 3A). The volatile compounds in the SST-155 °C-10 s, SST-170 °C-10 s, SST-190 °C-5 s groups were mainly aldehydes and heterocyclics (Figure 3B–D), but the relative content of aldehydes and heterocyclics in the SST-190 °C-5 s group was lower than other SST groups. Among them, hexanal in the aldehydes has the taste of grass and animal fat, which was a first-class product of auto-oxidation of linoleic acid. When their concentration increases, it seriously affects the flavor of food [53]. The 2-pentylfuran in the heterocyclic ring is generated by the cleavage of linoleic acid and has the flavor of nutty, beany, and buttery [54]. Because of its low threshold and great contribution to bad odor, hexanal and 2-pentylfuran were selected as the marker products of lipid oxidation [26,45].

**Figure 4:**
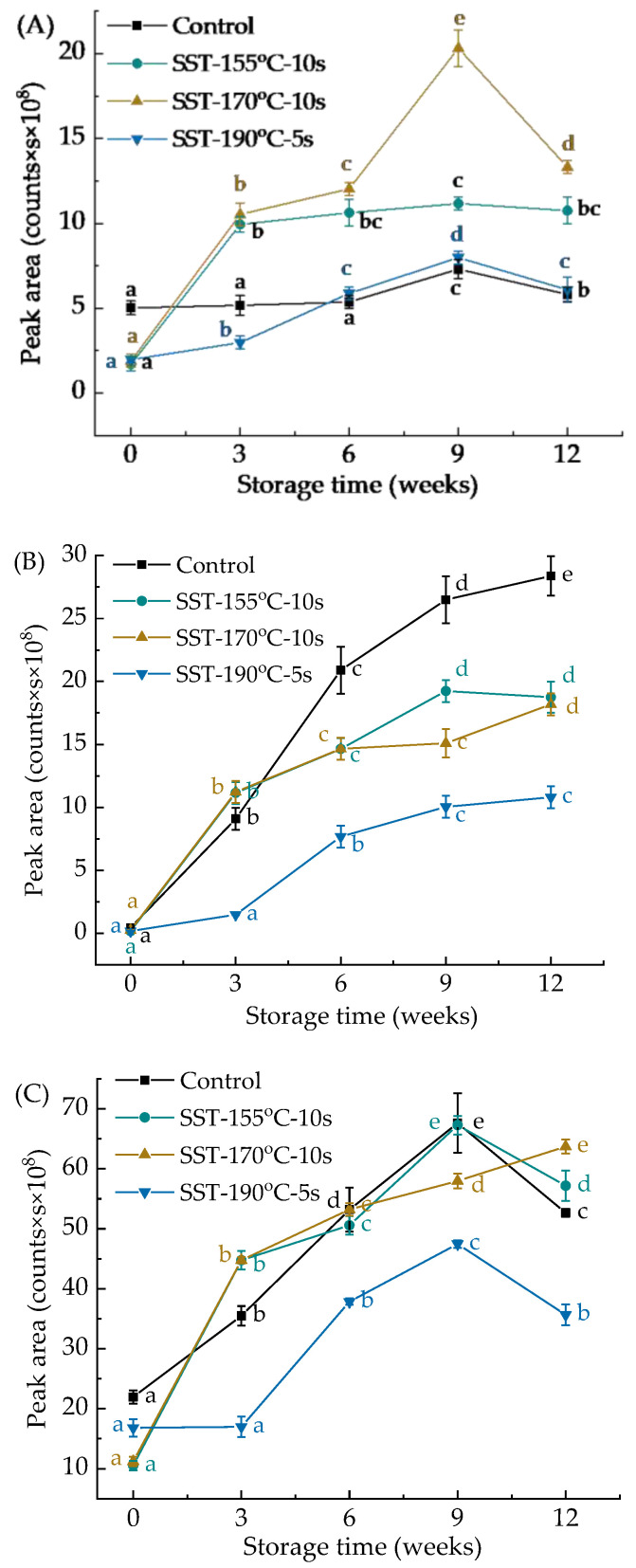
Effect of superheated steam treatment on peak area of volatile compounds in dried whole wheat noodles during storage. (**A**) The peak area of Hexanal, (**B**) the peak area of 2-pentylfuran, (**C**) total volatile compounds. The lowercase letters in the column chart indicate the significant difference of the same sample in different storage time at *p* < 0.05. SST: superheated steam treatment.

The contents of hexanal, 2-pentylfuran, and total volatile compounds in all sample groups gradually increased during storage (Figure 4). As shown in Figure 4A,B, during storage, the content of hexanal in the SST group was higher than that of the control group, and the content of 2-pentylfuran was lower than that of the control group. After 12 weeks of storage, the content of hexanal in the SST-155 °C-10 s, SST-170 °C-10 s, SST-190 °C-5 s group was 1.9, 2.3, 1-fold compared to the control group (Figure 4A), the content of 2-pentylfuran was 0.7, 0.6, and 0.4-fold that of the control group, respectively (Figure 4B). Hexanal was mainly produced by the cleavage of 13-hydroperoxide (13-ROOH) by the auto-oxidation of UFAs, and 2-pentylfuran was mainly produced by the cleavage of 9-ROOH by the enzymatic oxidation of UFAs [26,45,55]. LOX was conducive to the formation of 9-ROOH, the amount of 9-ROOH produced was over 7-fold that of 13-ROOH when the LOX was active, while the ratio of these two hydroperoxides in auto-oxidation was equivalent [45,56]. This indicated that LOX was passivated after the SST, the enzymatic oxidation pathway was inhibited and the auto-oxidation pathway was promoted. However, the enzymatic oxidation pathway of the SST-190 °C-5 s group was inhibited while the auto-oxidation pathway was not significantly different from the control group. In addition, the total volatile compounds in the SST groups were lower than the control group at the initial stage of storage. After 12 weeks of storage, the total volatile compounds of the SST-190 °C-5 s group was lower than that of the SST-155 °C-10 s, SST-170 °C-10 s group and control group. Besides, the total compounds of the SST-190 °C-5 s group was only 0.7-fold compared to the control group (Figure 4C). This indicated that the SST can significantly inhibit the generation of volatile compounds during the process and storage of DWWN, and the SST-190 °C-5 s condition was the most effective.

## 4. Conclusions

The SST significantly inhibited the lipid hydrolysis and oxidation during the process and storage of DWWN. Compared with the control group of DWWN, the lipase and POD in the SST groups were completely passivated, and the LOX activity was reduced by more than 67.6%. During storage, the activities of lipase, LOX and POD were not detected in the SST-processed DWWN. The SST significantly inhibited the increase of fatty acid value, the degradation of UFAs, the consumption of tocols, and the generation of volatile compounds during the process and storage of DWWN. Among them, SST-190 °C-5 s had the best inhibitory effect. In addition, the SST might promote auto-oxidation while inhibiting the enzymatic hydrolysis and enzymatic oxidation pathways, which depended on the treatment time of the SST. Therefore, SST is expected to be a method to improve the lipid stability of whole wheat products.

## Figures and Tables

**Table 1:** Effect of superheated steam treatment on lipase activity of dried whole wheat noodles during storage.

Storage Time (Weeks)	Lipase Activity (U/g·min^−1^)			
	Control	SST-155 °C-10 s	SST-170 °C-10 s	SST-190 °C-5 s
0	46.60 ± 2.88 ^a,^*	-	-	-
3	34.11 ± 2.88 ^b^	-	-	-
6	28.70 ± 1.29 ^b,c^	-	-	-
9	24.40 ± 0.96 ^c,d^	-	-	-
12	21.21 ± 1.76 ^d^	-	-	-

* The superscript lowercase letters indicate the significant difference of the same sample in different storage time at *p* < 0.05. SST: superheated steam treatment, -: the absorbance of the reaction system did not change within 3 min.

**Table 2:** Effect of superheated steam treatment on lipoxygenase activity of dried whole wheat noodles during storage.

Storage Time (Weeks)	Lipoxygenase Activity (U/g·min^−1^)			
	Control	SST-155 °C-10 s	SST-170 °C-10 s	SST-190 °C-5 s
0	574.43 ± 35.32 ^a,^*	186.18 ± 20.76	176.17 ± 20.76	164.84 ± 7.06
3	217.87 ± 2.83 ^b^	-	-	-
6	51.97 ± 3.46 ^c^	-	-	-
9	49.83 ± 0.00 ^c^	-	-	-
12	39.98 ± 0.00 ^c^	-	-	-

* The superscript lowercase letters indicate the significant difference of the same sample in different storage time at *p* < 0.05. SST: superheated steam treatment, -: the absorbance of the reaction system did not change within 3 min.

**Table 3:** Effect of superheated steam treatment on peroxidase activity of dried whole wheat noodles during storage.

Storage Time (Weeks)	Peroxidase Activity (U/g·min^−1^)			
	Control	SST-155 °C-10 s	SST-170 °C-10 s	SST-190 °C-5 s
0	1412.36 ± 66.66 ^a,^*	-	-	-
3	668.21 ± 43.90 ^b^	-	-	-
6	579.85 ± 61.01 ^b,c^	-	-	-
9	514.56 ± 18.61 ^c^	-	-	-
12	583.79 ± 29.07 ^b,c^	-	-	-

* The superscript lowercase letters indicate the significant difference of the same sample in different storage time at *p* < 0.05. SST: superheated steam treatment, -: the absorbance of the reaction system did not change within 3 min.

**Table 4:** Effect of superheated steam treatment on fatty acid composition of dried whole wheat noodles during storage (mg/g flour, mean ± standard deviation, *n* = 3).

Sample/Storage Time (Weeks)	Fatty Acid Composition (mg/g)							
	Palmitic Acid (C16:0)	Stearic Acid (C18:0)	Oleic Acid (C18:1)	Linoleic Acid (C18:2)	Linolenic Acid (C18:3)	Total SFAs	Total UFAs	Total FFAs
Control								
0	2.24 ± 0.01 ^A,a,^*	0.65 ± 0.00 ^A,a^	2.62 ± 0.00 ^B,a^	5.90 ± 0.01 ^C,a^	1.31 ± 0.00 ^B,a^	2.89 ± 0.01 ^A,a^	9.83 ± 0.02 ^B,a^	12.72 ± 0.03 ^B,a^
3	2.17 ± 0.05 ^B,b,c^	0.63 ± 0.00 ^B,b^	2.60 ± 0.05 ^B,a^	5.72 ± 0.05 ^C,b^	1.31 ± 0.01 ^B,a^	2.80 ± 0.05 ^B,b^	9.63 ± 0.04 ^C,b^	12.43 ± 0.03 ^C,b^
6	2.21 ± 0.03 ^A,a,b^	0.64 ± 0.01 ^A,b^	2.58 ± 0.10 ^A,a^	5.14 ± 0.03 ^B,c^	1.31 ± 0.02 ^B,a^	2.84 ± 0.03 ^A,a,b^	9.04 ± 0.09 ^B,c^	11.88 ± 0.12 ^B,c^
9	2.15 ± 0.02 ^D,b,c^	0.53 ± 0.00 ^B,c^	2.45 ± 0.01 ^D,b^	5.13 ± 0.05 ^D,c^	1.09 ± 0.00 ^C,b^	2.68 ± 0.02 ^D,c^	8.67 ± 0.06 ^D,d^	11.34 ± 0.09 ^D,d^
12	2.13 ± 0.03 ^D,c^	0.52 ± 0.01 ^C,c^	2.42 ± 0.04 ^C,b^	5.01 ± 0.03 ^D,d^	1.07 ± 0.02 ^C,b^	2.66 ± 0.02 ^D,c^	8.50 ± 0.03 ^D,e^	11.16 ± 0.05 ^D,e^
SST-155 °C-10 s								
0	2.27 ± 0.02 ^A,a^	0.65 ± 0.00 ^A,a^	2.77 ± 0.01 ^A,a^	7.76 ± 0.06 ^A,B,a^	1.42 ± 0.01 ^A,a^	2.92 ± 0.02 ^A,a^	11.95 ± 0.07 ^A,a^	14.87 ± 0.10 ^A,a^
3	2.23 ± 0.05 ^B,a^	0.64 ± 0.00 ^B,b^	2.69 ± 0.06 ^A,B,b^	7.43 ± 0.22 ^A,B,a,b^	1.39 ± 0.01 ^A,b^	2.87 ± 0.06 ^B,a^	11.51 ± 0.28 ^A,B,b^	14.38 ± 0.34 ^B,a,b^
6	2.25 ± 0.09 ^A,a^	0.64 ± 0.01 ^A,a,b^	2.68 ± 0.07 ^A,b,c^	7.13 ± 0.36 ^A,B,b^	1.37 ± 0.02 ^A,B,b^	2.89 ± 0.10 ^A,a^	11.18 ± 0.45 ^A,B,b^	14.07 ± 0.55 ^A,B,b^
9	2.20 ± 0.03 ^C,a^	0.53 ± 0.00 ^B,c^	2.60 ± 0.05 ^C,c,d^	7.40 ± 0.13 ^C,b^	1.16 ± 0.01 ^B,c^	2.73 ± 0.04 ^C,b^	11.16 ± 0.17 ^C,b^	13.89 ± 0.21 ^C,b^
12	2.22 ± 0.00 ^B,a^	0.53 ± 0.00 ^B,c^	2.59 ± 0.00 ^B,d^	7.39 ± 0.04 ^B,b^	1.16 ± 0.00 ^B,c^	2.74 ± 0.00 ^B,b^	11.14 ± 0.04 ^B,b^	13.89 ± 0.04 ^B,b^
SST-170 °C-10 s								
0	2.32 ± 0.05 ^A,a^	0.65 ± 0.00 ^A,a^	2.83 ± 0.06 ^A,a^	7.33 ± 0.10 ^B,a,b^	1.43 ± 0.02 ^A,a^	2.97 ± 0.05 ^A,a^	11.58 ± 0.16 ^A,a,b^	14.55 ± 0.21 ^A,a^
3	2.27 ± 0.03 ^B,a,b^	0.63 ± 0.00 ^B,b^	2.66 ± 0.08 ^A,B,a,b^	7.31 ± 0.39 ^B,a,b^	1.39 ± 0.03 ^A,b^	2.90 ± 0.03 ^B,a,b^	11.35 ± 0.50 ^B,a,b^	14.26 ± 0.51 ^B,a,b^
6	2.28 ± 0.13 ^A,a,b^	0.65 ± 0.01 ^A,a^	2.69 ± 0.10 ^A,b^	7.17 ± 0.44 ^A,b^	1.37 ± 0.03 ^A,b^	2.93 ± 0.14 ^A,a,b^	11.24 ± 0.58 ^A,a,b^	14.16 ± 0.72 ^A,a,b^
9	2.31 ± 0.02 ^B,a^	0.53 ± 0.00 ^B,c^	2.68 ± 0.00 ^B,a,b^	7.78 ± 0.07 ^B,a^	1.19 ± 0.01 ^A,c^	2.84 ± 0.02 ^B,b^	11.65 ± 0.07 ^B,a^	14.49 ± 0.09 ^B,a^
12	2.18 ± 0.02 ^C,b^	0.53 ± 0.00 ^B,c^	2.56 ± 0.01 ^B,b^	7.24 ± 0.05 ^C,b^	1.16 ± 0.01 ^B,d^	2.71 ± 0.02 ^C,c^	10.96 ± 0.07 ^C,b^	13.67 ± 0.08 ^C,b^
SST-190 °C-5 s								
0	2.30 ± 0.09 ^A,b^	0.64 ± 0.01 ^A,a^	2.78 ± 0.12 ^A,b^	7.95 ± 0.53 ^A,a,b^	1.43 ± 0.03 ^A,a^	2.94 ± 0.11 ^A,c^	12.15 ± 0.68 ^A,a^	15.10 ± 0.79 ^A,a^
3	2.38 ± 0.07 ^A,b^	0.64 ± 0.01 ^A,a^	2.77 ± 0.07 ^A,b^	7.86 ± 0.25 ^A,a,b^	1.41 ± 0.01 ^A,a,b^	3.03 ± 0.08 ^A,b,c^	12.05 ± 0.33 ^A,a^	15.08 ± 0.41 ^A,a^
6	2.42 ± 0.01 ^A,b^	0.65 ± 0.00 ^A,a^	2.79 ± 0.01 ^A,b^	7.70 ± 0.01 ^A,a,b^	1.40 ± 0.01 ^A,b^	3.07 ± 0.01 ^A,b^	11.90 ± 0.00 ^A,a^	14.97 ± 0.01 ^A,a^
9	2.41 ± 0.01 ^A,b^	0.55 ± 0.01 ^A,b^	2.75 ± 0.01 ^A,b^	8.23 ± 0.06 ^A,a^	1.20 ± 0.00 ^A,d^	2.96 ± 0.01 ^A,b,c^	12.19 ± 0.05 ^A,a^	15.15 ± 0.07 ^A,a^
12	2.48 ± 0.00 ^A,a^	0.55 ± 0.00 ^A,b^	3.05 ± 0.02 ^A,a^	7.51 ± 0.02 ^A,b^	1.26 ± 0.01 ^A,c^	3.03 ± 0.00 ^A,a^	11.82 ± 0.01 ^A,a^	14.85 ± 0.01 ^A,a^

* Superscript uppercase and lowercase letters indicate significant differences within rows and columns at *p* < 0.05, respectively. SST-superheated steam treatment, SFAs: saturated fatty acids, UFAs: unsaturated fatty acids, FFAs: free fatty acids.

**Table 5:** Effect of superheated steam treatment on the content of tocopherols and tocotrienols (μg/g) in dried whole wheat noodles during storage (mg/g flour, mean ± standard deviation, *n* = 3).

Sample	Storage Time (Weeks)	Tocopherols and Tocotrienols (μg/g)								
		α-Tocopherol	α-Tocotrienol	β-Tocopherol	β-Tocotrienol	γ-Tocotrienol	δ-Tocotrienol	Tocopherols	Tocotrienols	Tocols
	0	3.11 ± 0.12	0.59 ± 0.03	0.53 ± 0.05	1.89 ± 0.22	1.24 ± 0.10	0.37 ± 0.00	3.64 ± 0.08 ^B,a,^*	4.09 ± 0.35 ^B,a^	7.73 ± 0.28 ^B,a^
	3	1.92 ± 0.18	0.40 ± 0.01	0.54 ± 0.03	1.53 ± 0.19	0.62 ± 0.13	0.15 ± 0.03	2.47 ± 0.21 ^B,b^	2.70 ± 0.11 ^C,b^	5.16 ± 0.32 ^C,b^
Control	6	2.04 ± 0.08	0.43 ± 0.01	0.37 ± 0.01	1.80 ± 0.02	0.41 ± 0.01	nd	2.41 ± 0.09 ^D,b^	2.63 ± 0.01 ^D,b^	5.04 ± 0.10 ^D,b,c^
	9	1.68 ± 0.27	0.48 ± 0.03	0.51 ± 0.04	1.48 ± 0.14	0.36 ± 0.03	0.23 ± 0.03	2.19 ± 0.25 ^C,b,c^	2.55 ± 0.12 ^C,b^	4.74 ± 0.13 ^C,c,d^
	12	1.25 ± 0.03	0.81 ± 0.01	0.62 ± 0.10	1.43 ± 0.08	0.43 ± 0.00	nd	1.86 ± 0.13^C,c^	2.67 ± 0.07 ^C,b^	4.53 ± 0.15 ^C,d^
	0	2.77 ± 0.27	0.62 ± 0.20	0.98 ± 0.10	4.53 ± 0.68	1.45 ± 0.78	0.37 ± 0.04	3.75 ± 0.28^B,a^	6.98 ± 1.68 ^A,a^	10.72 ± 1.71 ^A,a^
	3	1.52 ± 0.02	0.37 ± 0.01	0.68 ± 0.12	2.60 ± 0.62	0.53 ± 0.05	0.18 ± 0.02	2.20 ± 0.10 ^A,B,c^	3.68 ± 0.66 ^C,b^	5.87 ± 0.75 ^C,b^
SST-	6	2.13 ± 0.06	1.03 ± 0.05	0.53 ± 0.08	1.51 ± 0.02	0.45 ± 0.01	nd	2.67 ± 0.05 ^C,b^	3.00 ± 0.02 ^C,b^	5.67 ± 0.06 ^C,b^
155 °C-10s	9	2.17 ± 0.27	0.37 ± 0.04	0.34 ± 0.02	1.31 ± 0.05	0.27 ± 0.00	0.22 ± 0.02	2.50 ± 0.25 ^B,C,b^	2.16 ± 0.02 ^C,b^	4.66 ± 0.23 ^C,b^
	12	1.73 ± 0.02	0.47 ± 0.00	0.63 ± 0.03	1.46 ± 0.08	0.32 ± 0.00	nd	2.36 ± 0.01 ^B,b,c^	2.25 ± 0.08 ^D,b^	4.61 ± 0.09 ^C,b^
	0	2.88 ± 0.19	0.48 ± 0.02	1.35 ± 0.12	6.46 ± 0.66	0.55 ± 0.06	0.33 ± 0.01	4.24 ± 0.26 ^A,a^	7.47 ± 0.77 ^A,a^	12.05 ± 0.78 ^A,a^
	3	1.34 ± 0.04	0.56 ± 0.16	0.74 ± 0.03	3.67 ± 0.55	1.13 ± 0.16	0.13 ± 0.00	2.08 ± 0.07 ^C,d^	5.48 ± 0.55 ^B,b^	7.56 ± 0.47 ^B,b^
SST-	6	2.59 ± 0.08	1.02 ± 0.07	0.51 ± 0.12	1.74 ± 0.02	0.47 ± 0.02	nd	3.10 ± 0.18 ^B,b^	3.23 ± 0.06 ^B,c^	6.33 ± 0.15 ^B,c^
170 °C-10s	9	2.23 ± 0.23	0.32 ± 0.03	0.54 ± 0.02	2.31 ± 0.02	0.30 ± 0.05	0.24 ± 0.01	2.77 ± 0.25 ^B,c^	3.17 ± 0.01 ^B,c^	5.94 ± 0.26 ^B,c^
	12	1.78 ± 0.02	1.16 ± 0.10	0.66 ± 0.03	1.68 ± 0.05	0.47 ± 0.02	nd	2.44 ± 0.04 ^B,c,d^	3.31 ± 0.16 ^B,c^	5.75 ± 0.13 ^B,c^
	0	2.97 ± 0.10	0.53 ± 0.03	1.28 ± 0.03	5.94 ± 0.12	1.00 ± 0.19	0.36 ± 0.01	4.25 ± 0.07 ^A,a^	7.83 ± 0.16 ^A,b^	12.08 ± 0.18 ^A,a^
	3	1.66 ± 0.01	1.13 ± 0.01	1.56 ± 0.01	5.70 ± 0.03	1.33 ± 0.03	0.29 ± 0.01	3.22 ± 0.02 ^A,d^	8.45 ± 0.08 ^A,a^	11.67 ± 0.07 ^A,b^
SST-	6	2.53 ± 0.04	0.99 ± 0.01	1.29 ± 0.05	5.95 ± 0.16	0.40 ± 0.04	nd	3.82 ± 0.09 ^A,b^	7.33 ± 0.15 ^A,c^	11.15 ± 0.22 ^A,c^
190 °C-5s	9	2.40 ± 0.01	1.11 ± 0.04	1.17 ± 0.01	5.64 ± 0.01	0.26 ± 0.02	0.31 ± 0.02	3.57 ± 0.02 ^A,c^	7.33 ± 0.04 ^A,c^	10.90 ± 0.04 ^A,c,d^
	12	1.75 ± 0.02	1.09 ± 0.02	1.92 ± 0.04	5.47 ± 0.03	0.54 ± 0.07	nd	3.67 ± 0.06 ^A,c^	7.09 ± 0.05 ^A,d^	10.76 ± 0.08 ^A,d^

* Superscript uppercase and lowercase letters indicate significant differences within rows and columns at *p* < 0.05, respectively. SST: superheated steam treatment, nd: not detected, Tocols: the sum of tocopherols and tocotrienols.

## Data Availability

The data that support the findings of this study are available on request from the corresponding author. The data are not publicly available due to privacy or ethical restrictions.
